# The double penalty of the coronavirus: Decidedly this virus has not yet revealed all its secrets!

**DOI:** 10.1192/j.eurpsy.2022.1319

**Published:** 2022-09-01

**Authors:** R. Jomli, H. Jemli, H. Ghabi, U. Ouali, Y. Zgueb

**Affiliations:** 1 Razi Hospital, Psychiatry A, manouba, Tunisia; 2 university of tunis elmanar, Faculty Of Medicine Of Tunis, manouba, Tunisia

**Keywords:** affective disorder, COVID19, Suicide, hallucinations

## Abstract

**Introduction:**

The direct and indirect effects of the COVID-19 pandemic on the mental health of the population have become a concern in the field of research in psychiatry. First psychotic episodes following infection with SARS cov2 have been reported.

**Objectives:**

Through a clinical case, we will illustrate the association of psychiatric symptoms with SARS cov2 infection.

**Methods:**

We discussed , through a clinical case, the association of psychiatric symptoms with infection by the coronavirus 19.

**Results:**

L.R, Tunisian 52-year-old, diabetic (type 2) women, with no personal or family psychiatric history and no toxic habits. she did not receive receive covid 19 vaccination. Twenty days before her admission to the psychiatry departement, she had fever, cough, myalgia, and anosmia .The diagnosis of a SARS COv2 infection was retained by her general practitioner. Two weeks later she suddenly presented a persecutory delirium, distressing auditory hallucinations, and attempted rat poison suicide. On admission, The patient had a delirium of persecution towards her entourage and an auditory hallucinatory syndrome with distressing content. She was put on 1 mg of Risperidone with restitution ad integrum after 7 days. COVID-19 serology test detected IgM antibodies which allowed us to conclude that the symptomatology was related to the infection by this virus. For the etiological research, we performed a serology that confirmed the recent exposure to SARS COV2 and. The diagnosis retained is a brief psychotic disorder post-Sars Cov2.

**Conclusions:**

The advanced hypothesis that infection with SARS CoV-2 could be the cause of the psychiatric manifestations remains unclear to this day.

**Disclosure:**

No significant relationships.

